# Effects of Heat-Treated *Lactobacillus helveticus* CP790-Fermented Milk on Gastrointestinal Health in Healthy Adults: A Randomized Double-Blind Placebo-Controlled Trial

**DOI:** 10.3390/nu16142191

**Published:** 2024-07-10

**Authors:** Reiko Tanihiro, Masahiro Yuki, Katsuhisa Sakano, Masaki Sasai, Daisuke Sawada, Shukuko Ebihara, Tatsuhiko Hirota

**Affiliations:** 1 Core Technology Laboratories, Asahi Quality and Innovations, Ltd., Moriya 302-0106, Japan; masahiro.yuki@asahi-qi.co.jp (M.Y.); katsuhisa.sakano@asahi-qi.co.jp (K.S.); masaki.sasai@asahi-qi.co.jp (M.S.); daisuke.sawada@asahi-qi.co.jp (D.S.); tatsuhiko.hirota@asahi-qi.co.jp (T.H.); 2 Chiyoda Paramedical Care Clinic, Tokyo 101-0047, Japan; info@cpcc.co.jp

**Keywords:** *Lactobacillus helveticus*, postbiotic, gut microbiota, constipation, mood state

## Abstract

Probiotic-fermented milk is commonly used to maintain intestinal health. However, the effects of heat-treated fermented milk, which does not contain live microorganisms, on intestinal function are not yet fully understood. This study aimed to investigate whether heat-treated *Lactobacillus helveticus* CP790-fermented milk affects fecal microbiota and gut health as a “postbiotic”. A randomized, double-blind, placebo-controlled trial was conducted in healthy Japanese individuals aged 20–59 years with a tendency toward constipation. Participants consumed 100 mL of either the test beverage (*n* = 60) or placebo beverage (*n* = 60) for four weeks. The test beverages were prepared with heat-treated CP790-fermented milk, while the placebo beverages were prepared with nonfermented milk flavored with lactic acid. Fecal samples were analyzed using 16S rRNA gene sequencing. Constipation symptoms were assessed using defecation logs and the Patient Assessment of Constipation Symptoms (PAC-SYM) questionnaire. Mood state was also assessed using the Profile of Mood States 2 (POMS2) questionnaire to explore its potential as a “psychobiotic”. *Desulfobacterota* were significantly decreased by CP790-fermented milk intake. PICRUSt2 analysis predicted a decrease in the proportion of genes involved in the sulfate reduction pathway following the consumption of CP790-fermented milk. The CP790-fermented milk intervention significantly improved stool consistency and straining during defecation. These improvements were correlated with a decrease in *Desulfobacterota*. After the intervention, overall mood, expressed as total mood disturbance, and depression–dejection were significantly better in the CP790 group than in the placebo group. These results suggest that the intake of CP790-fermented milk could be effective in modulating gut microbiota and improving constipation symptoms and mood states.

## 1. Introduction

More than 100 trillion microorganisms live in the human intestinal tract and maintain a symbiotic relationship with their host, thereby maintaining homeostasis [1]. Disruption of homeostasis (dysbiosis) can be directly linked to the risk of various diseases [2]. Functional constipation is one of the most frequent bowel diseases, afflicting approximately 14% of the adult population worldwide [3]. Common symptoms of constipation include decreased frequency of defecation, hard stools, straining, feeling of incomplete evacuation, and abdominal discomfort [4]. Functional constipation is not life-threatening but has a significant negative impact on quality of life [5]. A recent cross-sectional study showed that functional constipation affects both absenteeism and presenteeism, resulting in decreased work productivity [6]. In other words, alleviating constipation is important not only from the perspective of health issues but also from the perspective of solving social issues. Fermented milk, one of the oldest probiotics, is often reported to be associated with reduced constipation [7]. Recent studies have shown that fermented milk has beneficial effects not only in reducing constipation, but also in modification of gut microbiota and many other aspects of physical health [8,9,10,11]. The fermented milk used in the present study was heat-treated and differed from the probiotic-fermented milk described above in that it did not contain any viable microorganisms. Postbiotics [12], defined in 2021 as “preparations of inanimate microorganisms and/or their components that confer a health benefit to the host”, are gaining attention due to the advantages associated with their lack of viable organisms. The advantages of postbiotics include the absence of the risk of bacterial translocation and the ease of application in terms of processing and distribution without restrictions on temperature or pH. However, few studies have examined the effects of heat-treated fermented milk as a postbiotic on human health. Heat-treated fermented milk has been reported to be effective in relieving rhinitis symptoms in patients with perennial allergic rhinitis [13] and in preventing infections in children [14], while its effects on gut health in healthy adults with a tendency toward constipation have not been reported. The heat-treated fermented milk used in this study was fermented with *Lactobacillus helveticus* CP790 (CP790). CP790 has a long food history, and fermented milk made with CP790 is used in beverages that have been manufactured and sold in the United States and Asia. Previous studies on CP790 have reported that it has strong proteolytic activity [15]. Furthermore, CP790-fermented milk has been reported to exert antihypertensive effects [16]. However, no previous reports exist on the relationship between CP790 and constipation or gut microbiota. In this study, we evaluated the effects of CP790 fermented milk on the intestinal microbiota, as well as on constipation symptoms in healthy adults with a tendency toward constipation, with the aim of determining whether CP790 fermented milk can function as a postbiotic. In addition, its effects on mood states were evaluated to explore its potential beneficial effects on mental health.

## 2. Materials and Methods

### 2.1. Study Design

**Figure 1:**
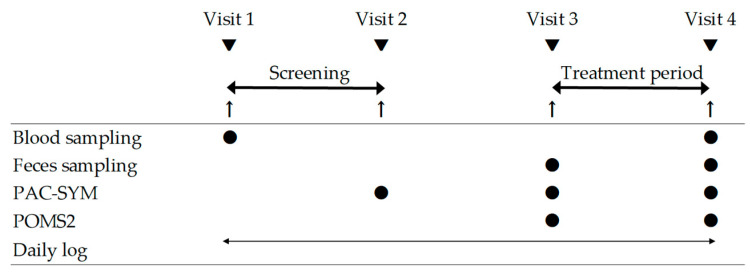
Schedule of this study. PAC-SYM, Patient Assessment of Constipation Symptoms; POMS2, Profile of Mood States 2.

This was a double-blind placebo-controlled parallel-group trial. The participants were recruited from the Chiyoda Paramedical Care Clinic (Tokyo, Japan). The participants visited the clinic four times, as shown in Figure 1: Visit 1 (beginning of the screening period), visit 2 (end of the screening period), visit 3 (beginning of the intervention), and visit 4 (end of the intervention). They were instructed to consume 100 mL of bottled water every morning from visits 1 to 3, followed by 100 mL of the test/placebo beverage instead of bottled water every morning from visits 3 to 4. Prior to the eligibility evaluation, screening was conducted, considering that bowel movements are altered by the ingestion of water contained in the test/placebo beverages. The subjects recorded their bowel movements for 28 days from visits 1 to 2, and those whose defecation frequency met the participation criteria, calculated based on their records from days 14 to 28, were included. Medical interviews, physical examinations, and physiological tests were performed during visits 1, 3, and 4. Blood sampling was performed at visits 1 and 4. Participants answered the Patient Assessment of Constipation Symptoms (PAC-SYM) questionnaire [17] at visits 2, 3, and 4. After visit 2, eligibility was determined based on the inclusion and exclusion criteria. Randomization and allocation were carried out by Evidence Marketing LLC (Tokyo, Japan). Participants were randomly allocated in a 1:1 ratio to either the CP790 or placebo group using blocked randomization based on age, sex, and defecation frequency. The CP790 or placebo group was blinded from investigators, evaluator, physician, and participants until the end of the trial. Defecation records for two weeks before the intake of the test/placebo beverage were defined as baseline data. At visits 3 and 4, the participants answered questions about their mood states, and fecal samples were collected. Participants were instructed to maintain daily records of bowel movements, consumption of the test/placebo beverages, and their physical condition. The participants were instructed to avoid supplements and health foods and maintain their lifestyle. This study was conducted between January and June 2023. The primary outcome was defined as changes in fecal microbiota, and the secondary outcomes were defined as bowel habits, constipation symptoms, and mood states.

### 2.2. Participants

The sample size was estimated using G*Power [18]. Based on a previous study [19] and assuming an effect size of 0.54, an alpha of 0.05, and a power of 0.80, the minimum sample size was estimated to be 55 per group. Therefore, the target number of participants was set to 120 (60 per group), assuming a dropout rate of approximately 10%. The key inclusion criteria were as follows: (1) Healthy Japanese adults between the ages of 20 and 59 years, (2) participants who have defecated at least three and fewer than six times per week, and (3) participants who provided written informed consent to participate in this study. The key exclusion criteria were as follows: (1) Participants with a history or current history of severe hepatic, cardiac, renal, or digestive disease; (2) participants with recurrent constipation and diarrhea; (3) participants who used gastrointestinal drugs at least once a week; (4) participants with loose stools from milk; (5) participants with a definite increase in defecation frequency after a continuous intake of 100 mL of bottled water; (6) participants with constipation and diarrhea symptoms during menstruation; (7) participants who received antibiotics during this clinical test; (8) participants who consume lactic acid drinks, oligosaccharide, dietary fiber, or health foods at least twice a week; (9) participants who consume yogurt at least three times a week; (10) participants with heavy smoking, excessive drinking habits, or disordered lifestyles; (11) participants who planned to go on a long or trip abroad after visit 1 and during this study; (12) participants with >30.0 kg/m^2^ of BMI; (13) participants with food allergies; (14) participants who do not have a habit of consuming milk at least once a week; (15) pregnant, possibly pregnant, or lactating women; (16) participants judged to be ineligible by the investigator for any other reasons.

### 2.3. Study Product

CP790 fermented milk was prepared by adding CP790 starter to skim milk and fermenting at 37 °C for 24 h. The fermented milk was heat-sterilized and the number of CP790 bacteria was determined and used in the test beverage. The test beverage was adjusted to contain 1 × 10^10^ CP790 counts per 100 mL. Taste was adjusted using sweeteners, and the beverage was sterilized. Placebo beverages were prepared by replacing fermented milk with skim milk and whey protein concentrate, and adding lactic acid to match the appearance, taste, nutritional content, and pH of the test beverages as much as possible. Live bacteria were not detected in the test or placebo beverages. The supplementary beverages were produced by Asahi Soft Drinks Co., Ltd. (Tokyo, Japan).

### 2.4. Fecal Microbiota

Fecal samples were collected at home in a container containing guanidine thiocyanate solution. The collected samples were stored at 4 °C before DNA isolation. The DNA was isolated using the bead-beating method. The fecal samples were pulverized using a ShakeMaster^®^ NEO (Bio Medical Science, Tokyo, Japan), left at 65 °C for 10 min, and centrifuged at 12,000× *g* for 2 min. Genomic DNA was extracted from the collected centrifugal supernatant using a Lab-Aid824s DNA Extraction Kit (ZEESAN, Xiamen, China). 16S rRNA gene (V3-V4) amplicon sequencing was performed by Bioengineering Lab. Co., Ltd. (Sagamihara, Japan). dsDNA libraries were prepared using a two-step tailed PCR method. A Bio Tek Synergy H1 plate reader (Agilent, Santa Clara, CA, USA) and QuantiFluor dsDNA System (Promega, Madison, WI, USA) were used to measure library concentrations. Library quality was evaluated using a Fragment Analyzer (Agilent) and a dsDNA 915 Reagent Kit (Agilent). Paired-end 2 × 300 bp sequencing on the Illumina MiSeq^®^ platform was performed using the MiSeq Reagent Kit v3 (Illumina, San Diego, CA, USA). The 16S rRNA gene sequencing data and metadata were deposited in the DDBJ BioProject database (accession number: PRJDB18202). The sequences of all samples were processed using QIIME2 (v.2022.11). The DADA was used to exclude chimeric and noise sequences. The taxonomic assignment of the sequences was accomplished using SILVA 138. The protocols for amplicon libraries have been described in previous studies [20,21]. Alpha diversity metrics (Shannon’s entropy, Chao1, Pielou’s evenness, Faith’s PD, and observed features) were calculated to evaluate microbiome richness and evenness among the CP790 and placebo groups. The groups were compared using the Kruskal–Wallis test. Beta diversity was determined using the unweighted UniFrac distance metric and principal coordinate analysis (PCoA). The groups were compared using PERMANOVA. Phylogenetic Investigation of Communities by Reconstruction of Unobserved States (PICRUSt2) [22] provided predictive functional profiling based on 16S rRNA gene amplicon analysis.

### 2.5. Bowel Habits, Stool Consistency, and Constipation Symptoms

The numbers of bowel movements, stool consistency, straining during defecation, and feeling of incomplete evacuation were recorded daily by the participants. Stool consistency was evaluated using the Bristol Stool Scale (BSS) on the following seven levels [23]: (1) separate hard lumps, similar to nuts; (2) sausage-shaped but lumpy; (3) similar to a sausage but with cracks on the surface; (4) similar to a banana, smooth and soft; (5) soft blobs with clear-cut edges; (6) fluffy pieces with ragged edges, a mushy stool; and (7) watery, no solid pieces, entirely liquid. “Straining during defecation” and “feeling of incomplete evacuation” were scored a four-point Likert scale: 1 = “none”, 2 = “little”, 3 = “some”, 4 = “a lot”. The participants completed the PAC-SYM questionnaire at the time of their visit regarding the severity of their constipation symptoms over the last two weeks. The 12-items PAC-SYM was divided into three subscales: abdominal, rectal, and stool symptoms. Items were scored on a five-point Likert scale: 0 = “no symptoms”, 1 = “mild”, 2 = “moderate”, 3 = “severe”, 4 = “very severe” [17].

### 2.6. Mood States

Subjective psychological symptoms were evaluated using the Japanese translation of the Profile of Mood States 2nd Edition-Adult Short (shortened version of POMS2; Kaneko Shobo Inc., Tokyo, Japan), which is widely used to assess both overall and distinct mood states [24,25,26]. The shortened version of POMS2 consists of 35 items rated on a five-point Likert scale (0–4) and assesses five negative mood scales (anger–hostility, confusion–bewilderment, depression–dejection, fatigue–inertia, and tension–anxiety) and two positive mood scales (vigor–activity and friendliness). The total mood disturbance (TMD) score, a measure of the overall mood state, was calculated by subtracting the vigor–activity score from the sum of the five negative mood scores.

### 2.7. In Vitro Assay

Fecal samples were collected using a BD BBL CultureSwab Plus (BD Biosciences, Franklin Lakes, NJ, USA). The samples were suspended in Gifu anaerobic medium (GAM) and subsequently filtered through a 100 µm filter under anaerobic conditions. An equal volume of PreserWell MPR (MPR, Miyagi, Japan) was added to the filtrate, which was immediately stored at −80 °C until use. Filtered fecal stocks were inoculated in GAM with 1% methionine and anaerobically incubated at 37 °C for 24 h. CP790-fermented milk, placebo, or water were added to the medium at a final concentration of 4% at the start of the incubation. After incubation, the cultures were centrifuged at 9000× *g* for 5 min. To measure hydrogen sulfide concentration of the collected supernatant, we used hydrogen sulfide highly selective fluorescent probe, HSip-1 regent [27,28]. The measurement method was modified from the manufacturer’s instructions (DOJINDO LABORATORIES, Kumamoto, Japan). The fluorescence of HSip-1 was measured with microplate reader (λ_ex_ = 491 nm, λ_em_ = 516 nm). The hydrogen sulfide ratios of the CP790-fermented milk and placebo-supplemented cultures were calculated from the hydrogen sulfide concentration in the water-supplemented culture.

### 2.8. Daily Food Intake and Safety

Daily food intake was investigated using a brief self-administered diet history questionnaire (BDHQ) [29]. Safety was assessed through diary records, interviews, physical examinations, and general blood test results. The incidence of adverse events was monitored in the participants who consumed the study beverages at least once. Body weight, pulse, and systolic and diastolic blood pressures were measured and interviewed at visits 1, 3, and 4. Body height was measured at visit 1. General blood tests were performed by BML, Inc. (Tokyo, Japan).

### 2.9. Ethics Committee

The protocol was approved by the Institutional Review Board of Chiyoda Paramedical Care Clinic (Tokyo, Japan) according to the principles of the Declaration of Helsinki (approval date: 16 December 2022; approval no. 22121601). The trial is registered in the University Hospital Medical Information Network (UMIN) Clinical Trial Registry as UMIN000050001 (date of registration: 10 January 2023). Written informed consent was obtained from all participants prior to their enrolment in the study.

### 2.10. Statistical Analysis

Statistical analyses were performed using the IBM SPSS Statistics 25 software (IBM Japan Ltd., Tokyo, Japan). Comparison of the relative abundance of gut bacterial phyla, defecation habits, and mood states were performed using the Student’s *t*-test or paired *t*-test. Comparison of constipation symptoms and hydrogen sulfide production were performed using the Mann–Whitney U-test or Wilcoxon signed-rank test. The Fisher’s exact test was used to compare sex distribution. Spearman’s correlation was used to assess the correlation between defecation habits and fecal microbiota composition. The correlation network was visualized using Cytoscape v3.9.1. Differences were considered statistically significant at *p* < 0.05.

## 3. Results

### 3.1. Demographics

**Figure 2:**
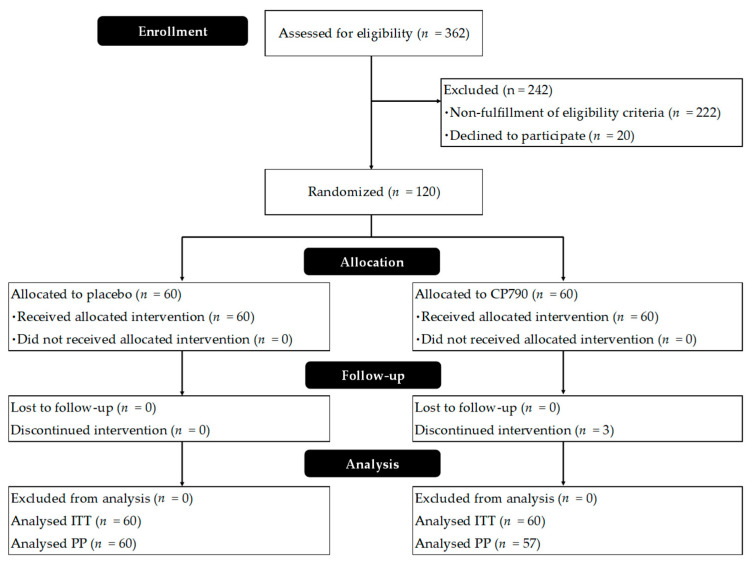
CONSORT flowchart.

In total, 120 participants were recruited according to the inclusion criteria and assigned to the two groups in a 1:1 ratio. Figure 2 shows a flow diagram of the study participants. Three subjects from the CP790 group exited the study for personal reasons, and 117 completed it. Finally, data from 117 subjects were used for the efficacy analysis. The safety analyses included 120 participants who had received the test beverage at least once. The baseline characteristics of participants who completed the study are shown in Table 1. No significant differences were found in age, sex, weight, height, BMI, defecation frequency, or stool consistency. High compliance was attained in this study; the frequencies of CP790 and placebo beverage consumption (%) were 99.94 ± 0.47 for CP790 and 100.00 ± 0.00 for placebo (mean ± standard deviation). The results of the BDHQ dietary survey showed no significant changes in dietary intake in either group.

### 3.2. Fecal Microbiota

**Figure 3:**
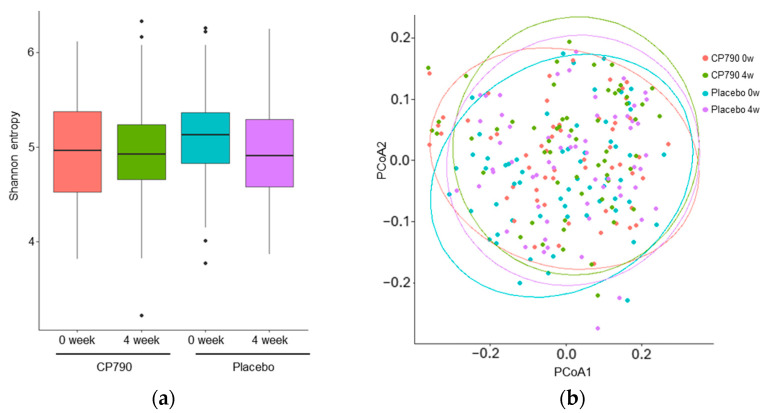
Microbial diversity analysis: (**a**) Shannon entropy; (**b**) principal coordinate analysis (PCoA) plot.

In this study, consumption of CP790-fermented milk reduced the *Desulfobacterota* abundance but did not change diversity. The alpha diversity metrics, including Shannon entropy, Chao1, Pielou’s evenness, Faith’s PD, and observed features, were not significantly different between CP790-fermented milk and placebo (Figure 3a and Figure S1). To compare the compositions of the microbial communities, the unweighted UniFrac distances were calculated (Figure 3b). There were no significant differences within and between groups.

To identify differences in taxonomic levels between the two groups, changes in the relative abundance of the taxonomic groups after the intervention were compared (Table 2). The relative abundance of *Desulfobacterota* was significantly reduced in the CP790 group compared to that in the placebo group (*p* = 0.036). Changes in the other major phyla, *Bacteroidota*, *Firmicutes*, *Proteobacteria*, and *Actinobacteriota*, were not significantly different between the groups.

### 3.3. Bowel Habits, Stool Consistency, and Constipation Symptoms

Ingestion of CP790-fermented milk improved stool consistency, straining during defecation, and abdominal symptoms related to constipation in this study. Table 3 shows changes in the defecation frequency, BSS, straining during defecation, and feeling of incomplete evacuation calculated from the defecation logs. At the baseline, no statistical differences in these parameters were observed (Table S1). The changes in the defecation frequency in the CP790-fermented group did not differ significantly from those in the placebo group. The BSS scores showed a significant increase in the CP790 group compared to the placebo group (*p* = 0.023). The consumption of CP790-fermented milk significantly reduced scores for straining during defecation compared to the placebo (*p* = 0.042). There was a trend toward a decrease in scores for feeling of incomplete evacuation in the CP790 group compared to the placebo group (*p* = 0.081).

Table 4 shows changes in scores based on the PAC-SYM questionnaire. At the baseline, no significant differences were found (Table S2). The changes in the total PAC-SYM score in the CP790 group were not significantly different from those in the placebo group. Although abdominal symptom scores showed a significant decrease in the CP790 group compared to the placebo group (*p* = 0.043), no significant differences were observed in terms of changes in the rectal and stool symptom scores between the groups.

### 3.4. Association between the Defecation Habits and Microbial Profile

**Figure 4:**
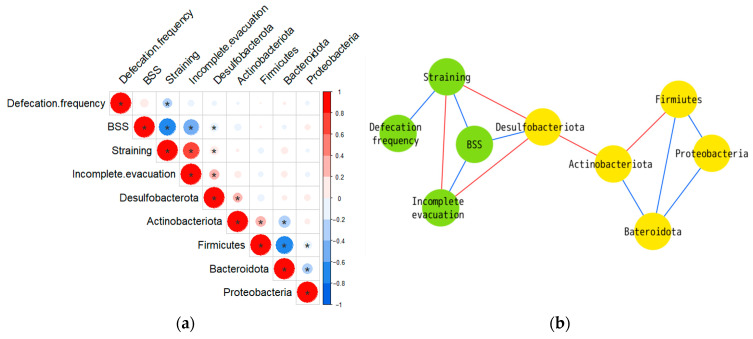
(**a**) Matrix of Spearman’s correlation coefficients. Color intensity indicates magnitude of correlation (* *p* < 0.05). Blue denotes a negative correlation and red denotes a positive correlation. The correlation coefficient values are presented in Table S3. (**b**) Correlation network. The network was visualized in Cytoscape. The nodes are microbiota (yellow) and bowel habits (green). Blue edges denote a significant negative and red edges denote a significant positive correlation.

The results of the correlation analyses are shown in Figure 4. Changes in the defecation frequency were not significantly associated with any of the phyla. A significant negative correlation was found between changes in the BSS scores and changes in *Desulfobacterota* (R = −0.188, *p* = 0.042). Changes in the scores for straining during defecation and feeling of incomplete evacuation were significantly and positively correlated with changes in *Desulfobacterota* (straining during defecation: R = 0.187, *p* = 0.043; feeling of incomplete evacuation: R = 0.225, *p* = 0.015). No other phyla besides *Desulfobacterota* showed a significant correlation with defecation phenotypes. The correlation coefficients are listed in Table S3.

### 3.5. PICRUSt2

**Figure 5:**
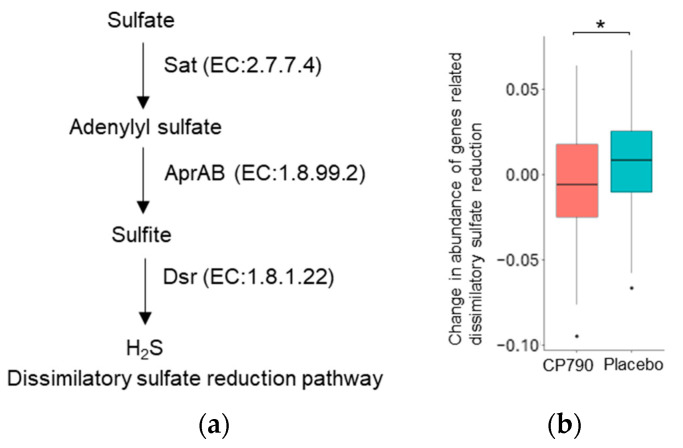
PICRUSt2 analysis: (**a**) Dissimilatory sulfate reduction pathway; (**b**) change in abundance of genes related dissimilatory sulfate reduction. Differences between groups were compared using the Student’s *t*-test (* *p* < 0.05). H_2_S: hydrogen sulfide.

To compare the abundance of hydrogen sulfide production-related genes between the two groups, we conducted PICRUSt2 analysis using the relative abundance of the 16S rRNA gene amplicon data. Because many hydrogen-sulfide-producing bacteria belong to *Desulfobacterota*, we calculated the abundance of dissimilatory sulfate reduction-related genes. The total abundance of these genes in the CP790 group was significantly decreased compared to that in the placebo group (*p* = 0.025) (Figure 5).

### 3.6. In Vitro Assay

**Figure 6:**
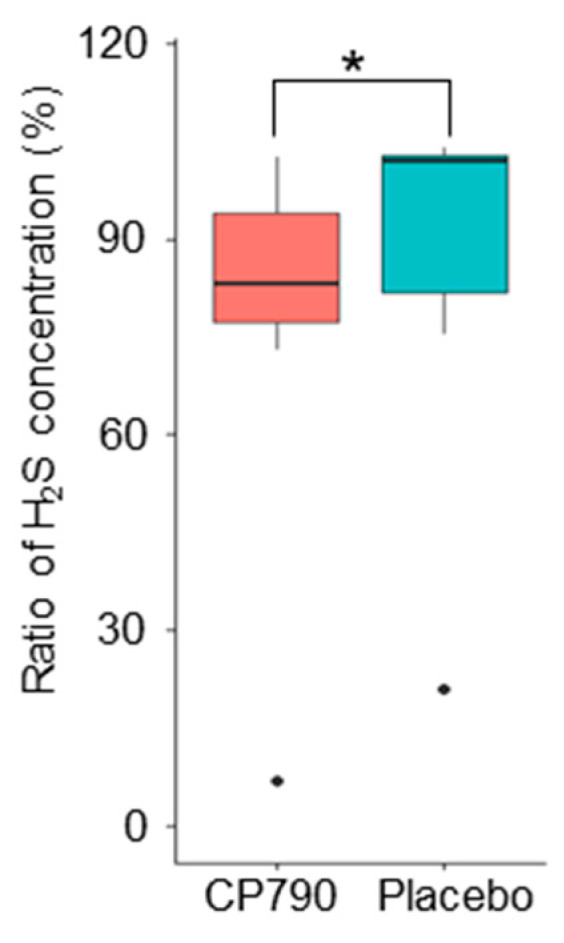
Ratio of hydrogen sulfide production. Data were analyzed using the Wilcoxon’s signed-rank test (* *p* < 0.05). H_2_S: hydrogen sulfide.

We investigated the inhibitory effect of CP790 on hydrogen sulfide production using an in vitro assay. The hydrogen sulfide concentrations of in vitro assay with fermented milk and placebo were 52.1 ± 12.8 and 60.5 ± 15.7 μM (mean ± standard error), respectively (Table 5). The ratio of hydrogen sulfide concentration in the culture supplemented with CP790 was significantly lower than that in the placebo (*p* = 0.043) (Figure 6). The in vitro study results did not contradict the predictions made from the results using PICRUSt2 analysis.

### 3.7. Mood States

In this study, administration of CP790-fermented milk improved the overall mood and depressed mood. The results of POMS2 for all subjects are shown in Table 6. Lower scores for TMD, anger–hostility, confusion–bewilderment, depression–dejection, fatigue–inertia, and tension–anxiety represent better conditions, while higher vigor–activity and friendliness scores represent better conditions. No statistically significant differences were found at baseline, except that the tension–anxiety score was significantly lower in the CP790 group than in the placebo group (*p* = 0.038). After intervention, the TMD score in the CP790 group was significantly lower than that in the placebo group (*p* = 0.046). Of the seven negative/positive mood scales, significantly lower depression–dejection scores were observed in the CP790 group than in the placebo group (*p* = 0.028), whereas the remaining six mood scales revealed no statistically significant differences between the groups. POMS2 is calculated as a standardized score based on the mean of a large sample of Japanese subjects. The majority of the participants in this study scored below 50, which is below the mean score of the Japanese population, and were in a relatively good mood state. Therefore, we conducted a subgroup analysis of participants with relatively poor mood states. Table 7 shows the results of POMS2 for subjects with poor mood states (*n* = 60), defined as a TMD median or above. The baseline tension–anxiety score was significantly lower in the CP790 group than in the placebo group (*p* = 0.025), whereas the remaining six mood scales and TMD scores did not differ statistically between the two groups at baseline. After the intervention, the TMD, depression–dejection, anger–hostility, fatigue–inertia, and tension–anxiety scores were significantly lower in the CP790 group than in the placebo group (*p* = 0.007, *p* = 0.050, *p* = 0.015, and *p* = 0.014, respectively), whereas no significant differences were found in confusion–bewilderment, vigor–activity, and friendliness scores between the groups.

### 3.8. Safety Analysis

Changes in the complete body composition, blood pressure, blood cell content, lipid parameters, and renal and liver functions were evaluated. No significant differences in these changes were found between the two groups. No severe or moderate adverse events were observed during the study period.

## 4. Discussion

In this study, the effects of *Lactobacillus helveticus* CP790-fermented milk on gut microbiota and defecation were examined. We also aimed to gain insights into the mechanisms by which CP790-fermented milk affects constipation symptoms by examining the relationship between changes in the intestinal microbiota and changes in defecation. No significant difference in the defecation frequency was observed between the two groups. The BSS scores were significantly increased from the baseline in the CP790 group (*p* < 0.001), but not in the placebo group (from 3.48 ± 0.09 to 3.87 ± 0.07 in the CP790 group, from 3.49 ± 0.09 to 3.64 ± 0.08 in the placebo group) (mean ± standard error). Furthermore, straining during defecation was significantly mitigated by CP790 intervention. It can be assumed that the consumption of CP790-fermented milk softened hard stools, bringing them closer to the ideal state (BSS score = 4), and allowing defecation with less straining. Constipation is often classified as either dyssynergic defecation, slow transit constipation, or normal transit constipation [30]. Satish et al. showed that patients with dyssynergic defecation have greater psychological distress and lower health-related quality of life (QOL) than those with slow transit constipation [31]. Namely, difficulty in defecation could be one of the most serious issues for constipation sufferers. Therefore, reducing straining during defecation is of great significance from the perspective of improving health-related QOL.

The participants were instructed to record the number of bowel movements, stool consistency, straining during defecation, and feeling of incomplete evacuation on their defecation logs every day, while they answered the PAC-SYM questionnaire at the clinic visit with reflection on constipation symptoms over the past two weeks. Changes in rectal symptoms, stool symptoms, and total PAC-SYM scores did not differ significantly between the groups. In contrast, the abdominal symptom scores were significantly decreased in the CP790 group compared with the placebo group. This suggests that the abdominal symptoms related to constipation could be ameliorated by supplementation with CP790-fermented milk. Therefore, CP790-fermented milk could be a beneficial postbiotic for normalizing stool consistency, reducing straining during defecation, and improving abdominal symptoms associated with constipation.

Gut microbiota analysis showed no significant differences between the CP790 and placebo groups in terms of both alpha and beta diversity. A comparison of the changes in abundance at the phylum level between the two groups showed a significant decrease in the phylum *Desulfobacterota* in the CP790 group compared to that in the placebo group. *Desulfobacterota* obtains energy in the process of reducing sulfate [32,33]. *Desulfobacterota* is not a direct pathogen and is present in healthy human colon; however, an increase in its abundance may lead to the development of ulcerative colitis and irritable bowel syndrome (IBS) [33,34]. Recently, *Desulfobacterota* was reported to be more abundant in the feces of IBS-constipation (IBS-C) patients than in healthy subjects [35].

In this study, CP790-fermented milk decreased the abundance of *Desulfobacterota* and increased the BSS score; these two parameters showed a significant negative correlation. In addition, straining during defecation and feeling of incomplete evacuation decreased after the CP790-fermented milk intervention, and both showed significant positive correlations with the abundance of *Desulfobacterota*. Interestingly, none of the phyla other than *Desulfobacterota* were correlated with these defecation habits, suggesting that *Desulfobacterota* may play an important role in the postbiotic action of CP790-fermented milk.

Therefore, we predicted the functional profiles of the gut microbiota from 16S rRNA metagenomic data and found a significant decrease in sulfate reduction reactions in the CP790 group compared to the placebo group. The final product of the sulfate reduction reaction is hydrogen sulfide, and *Desulfobacterota* is a well-known hydrogen-sulfide-producing bacterium [33]. Furthermore, in vitro assay confirmed that CP790-fermented milk reduced the production of hydrogen sulfide by intestinal bacteria.

Hydrogen sulfide is a known physiological mediator of inflammation that contributes to the development of inflammatory bowel diseases [36]. Furthermore, recent studies have shown that hydrogen sulfide enhances visceral pain by activating T-type calcium channels and transient receptor potential ankyrin-1 (TRPA1) [37]. Through these cascades, it has been reported that hydrogen sulfide produced in the colon is involved in colonic pain and colonic hypersensitivity, which are symptoms of IBS [38,39]. Abdominal pain is a component of the abdominal symptom score in the PAC-SYM questionnaire, which decreased after the CP790-fermented milk intervention. Although further research is needed to elucidate this, it is possible that the reduction in hydrogen sulfide production may be involved in the relief of abdominal symptoms related to constipation with CP790-fermented milk. To the best of our knowledge, few studies have been conducted on the ingredients that reduce colonic hydrogen-sulfide-producing bacteria and hydrogen sulfide production, and only one animal study has been reported to date. Deng et al. reported that oral administration of prebiotic inulin to pigs reduced enteric hydrogen-sulfide-producing bacteria and the emission of hydrogen sulfide gas [40]. As there is no precedent for this in postbiotics, CP790-fermented milk shows promise as a novel postbiotic that can reduce hydrogen-sulfide-producing bacteria.

Abnormal stress responses in germ-free mice and dysbiosis caused by chronic stress suggest a bidirectional relationship between mental health and the gut microbiota [41]. Approximately a decade ago, “psychobiotics” were defined as probiotics that confer mental health benefits to the host when consumed in a particular quantity through the interaction with commensal gut bacteria [42]. Recently, evolving the concept of psychobiotics to encompass exogenous bacteria-mediated effects on the brain, including postbiotics, has been proposed [43]. Therefore, to assess the potential of postbiotic CP790-fermented milk as a psychobiotic, we investigated its effects on mood states. The results of the assessment using POMS2 suggest that CP790-fermented milk may improve the overall mood state and depression–dejection, and that its effect may be more pronounced in subjects with poor mood states.

Two types of probiotic-fermented milk have been reported to function as psycobiotics [44,45]. *Lacticaseibacillus* fermented milk has been reported to alleviate depressive symptoms in patients with major depressive disorder or bipolar disorder [44], and *Bifidobacterium* fermented milk has been reported to decrease anxiety-related behaviors in rats [45]. Among postbiotics, recently, two heat-killed *Lactobacillus* bacterial bodies were reported to enhance “friendliness”, a positive mood, respectively [46,47] and another heat-killed *Lactobacillus* bacterial body was reported to improve trait anxiety, a negative mood [48]. Heat-killed *Lactobacillus* bacterial bodies may act on positive and negative mood states; however, the detailed mechanisms underlying their functioning remain unclear. *Desulfobacterota*, whose reduction was found after the heat-treated CP790-fermented milk intervention, produces lipopolysaccharide (LPS), which induces depressive symptoms through systemic inflammation [49,50,51]. A decrease in LPS-producing bacteria might play a key role for postbiotic CP790-fermented milk to improve overall and negative mood states.

Although this study is the first to report the effect of CP790-fermented milk on gut and mental health, it has several limitations. CP790-induced gut microbiota alterations may change the composition of gut metabolites that are closely related to the host physiological system; however, we did not include metabolomic analysis for detecting fecal metabolites. Combining the fecal metabolome with fecal microbiome may provide a better understanding of how CP790-fermented milk improves gut health. Additionally, this study assessed the effects of a 4-week test beverage ingestion, which is a relatively short intervention for a study evaluating the effects on mental health. Longer intervention trials are needed to more reliably assess the effects of test beverage administration on mood states. Moreover, a more pronounced improvement in mood states was found in the subgroup with poor mood states, but the sample size for subgroup analysis was small (*n* = 60). Further studies with larger cohorts are required to more precisely evaluate the benefits of test beverages in people with poor mood states. Finally, we assessed the effects on mood states using a questionnaire without any objective evaluation, such as saliva cortisol measurements. Further research, including objective evaluations, is needed to understand the mechanism of action of CP790-fermented milk in different mood states. Recently, *Desulfobacterota* was found to be closely associated with extraintestinal disorders, such as Parkinson’s disease in the elderly and autism spectrum disorders in children, through gut-derived hydrogen sulfide and LPS [52]. Therefore, findings on materials that can control *Desulfobacterota* abundance may help for the physical and mental health of a wide range of generations. In the future, we would like to further explore the relationship between CP790-fermented milk and *Desuolfobacterota* to develop new interventions for depression.

## 5. Conclusions

Four weeks of ingestion of *Lactobacillus helveticus* CP790-fermented milk could decrease *Desulfobacterota* abundance and improve stool consistency, straining during defecation, and abdominal symptoms related to constipation in healthy subjects. Additionally, it also could improve overall mood and depressed mood in healthy individuals. These results indicate that CP790-fermented milk may help improve the health of individuals with mental and physical distress. A novel finding of this study is that the consumption of heat-treated CP790-fermented milk decreases *Desulfobacterota* abundance. This finding has potential implications for understanding the gut–brain axis and the development of new interventions.

## Figures and Tables

**Table 1:** Baseline characteristics of the participants.

Items (Unit)	CP790 (*n* = 57)		Placebo (*n* = 60)		*p*-Values
	Mean	SE ^1^	Mean	SE ^1^	
Age (years)	46.9	1.3	46.3	1.2	0.756 ^2^
Male/female	36/21		38/22		1.000 ^3^
Height (cm)	166.3	6.6	167.1	8.3	0.583 ^2^
Weight (kg)	61.1	1.4	61.5	1.4	0.863 ^2^
Body mass index (kg/m^2^)	22.0	0.4	21.9	0.3	0.873 ^2^
Defecation frequency (times/week)	4.35	0.13	4.36	0.15	0.970 ^2^
Bristol Scale Score	3.48	0.09	3.49	0.09	0.942 ^2^

^1^ SE: standard error; ^2^ *p*-values calculated using the Student’s *t*-test; ^3^ *p*-value calculated using the Fisher’s exact test.

**Table 2:** Changes in the relative abundance in the phylum levels from the baseline.

Phyla (%)	CP790 (*n* = 57)		Placebo (*n* = 60)	
	Mean	SE ^1^	Mean	SE ^1^
*Bacteroidota*	1.05	1.29	2.94	1.19
*Firmicutes*	1.73	1.05	−1.20	1.08
*Proteobacteria*	−1.27	0.79	−1.02	0.69
*Actinobacteriota*	−0.23	0.23	−0.28	0.12
*Desulfobacterota* *	−0.51	0.14	−0.15	0.09

^1^ SE: standard error. Differences between the groups were compared using the Student’s *t*-test (* *p* < 0.05).

**Table 3:** Changes in the defecation habits from the baseline.

Items	CP790 (*n* = 57)		Placebo (*n* = 60)	
	Mean	SE ^1^	Mean	SE ^1^
Defecation frequency (times/week)	1.04	0.20	1.01	0.20
Bristol Scale Scores *	0.39	0.07	0.15	0.08
Straining during defecation *	−0.29	0.06	−0.11	0.06
Feeling of incomplete evacuation	−0.28	0.06	−0.14	0.05

^1^ SE: standard error. Differences between groups were compared using the Student’s *t*-test (* *p* < 0.05).

**Table 4:** Changes in the PAC-SYM scores from the baseline.

Items	CP790 (*n* = 57)		Placebo (*n* = 60)	
	Mean	SE ^1^	Mean	SE ^1^
Abdominal symptoms *	−0.14	0.05	−0.06	0.04
Rectal symptoms	−0.11	0.04	−0.15	0.05
Stool symptoms	−0.29	0.06	−0.19	0.05
Total PAC-SYM	−0.20	0.04	−0.14	0.04

^1^ SE: standard error. Differences between the groups were compared using the Mann–Whitney U-test (* *p* < 0.05).

**Table 5:** Hydrogen sulfide production in in vitro assays.

Conditions	Hydrogen Sulfide (µM)	
	Mean	SE ^1^
CP790	52.1	12.8
Placebo	60.5	15.7

^1^ SE: standard error.

**Table 6:** POMS2 scores in all subjects.

Items	Baseline				Post-Intervention			
	CP790 (*n* = 57)		Placebo (*n* = 60)		CP790 (*n* = 57)		Placebo (*n* = 60)	
	Mean	SE ^1^	Mean	SE ^1^	Mean	SE ^1^	Mean	SE ^1^
Total mood disturbance	41.67	0.78	43.80	0.93	41.19	0.66 *	43.65	1.01
Anger–hostility	43.46	0.77	44.93	1.04	42.74	0.49	44.78	1.07
Confusion–bewilderment	43.42	0.73	44.58	0.88	43.60	0.64	44.97	0.89
Depression–dejection	43.84	0.64	44.77	0.65	43.79	0.53 *	45.80	0.72
Fatigue–inertia	42.07	0.68	44.20	0.92	41.96	0.60	43.62	0.94
Tension–anxiety	42.89	0.76 *	45.37	0.89	42.28	0.82	44.45	0.92
Vigor–activity	54.93	1.23	53.32	1.11	55.65	1.25	53.88	1.21
Friendliness	54.32	1.34	53.02	1.13	54.75	1.47	52.77	1.26

^1^ SE: standard error. Differences between groups were compared using the Student’s *t*-test (* *p* < 0.05).

**Table 7:** POMS2 scores in subjects with poor mood state.

Items	Baseline				Post-Intervention			
	CP790 (*n* = 26)		Placebo (*n* = 34)		CP790 (*n* = 26)		Placebo (*n* = 34)	
	Mean	SE ^1^	Mean	SE ^1^	Mean	SE ^1^	Mean	SE ^1^
Total mood disturbance	46.15	1.08	48.59	0.99	44.62	0.79 *	48.53	1.15
Anger–hostility	46.58	1.33	48.88	1.48	44.42	0.73 *	48.76	1.55
Confusion–bewilderment	47.12	1.17	48.15	1.19	46.04	0.91	48.65	1.16
Depression–dejection	45.69	1.27	46.74	0.98	45.00	0.95 *	47.88	1.08
Fatigue–inertia	45.88	0.94	48.09	1.17	43.92	0.82 *	47.71	1.19
Tension–anxiety	46.12	1.07 *	49.47	0.99	44.88	0.88 *	48.41	1.07
Vigor–activity	49.81	1.06	48.79	0.96	49.81	1.16	49.18	1.19
Friendliness	49.19	1.43	49.06	1.14	47.69	1.56	49.09	1.33

^1^ SE: standard error. Differences between groups were compared using the Student’s *t*-test (* *p* < 0.05).

## Data Availability

The data used in this study are not publicly available but can be requested from the corresponding author.

## Supplementary Materials

The following supporting information can be downloaded at: https://www.mdpi.com/article/10.3390/nu16142191/s1, Figure S1: Alpha diversity metrics; Table S1: Defecation habits; Table S2: PAC-SYM scores; Table S3: Spearman’s correlation coefficients.
