# Supplementary material for: Effects of Heat-Treated Lactobacillus helveticus CP790-Fermented Milk on Gastrointestinal Health in Healthy Adults: A Randomized Double-Blind Placebo-Controlled Trial
Source: Nutrients. 2024 Jul 10;16(14):2191. doi: 10.3390/nu16142191 (PMC11280423; doi:10.3390/nu16142191)
Supplement: Supplementary file 1 [file nutrients-16-02191-s001.zip › nutrients-3096316-supplementary.pdf]

Supplementary Information

Effects of Heat-Treated *Lactobacillus helveticus* CP790-Fermented Milk on Gastrointestinal Health in Healthy Adults: A Randomized Double-blind Placebo-controlled Trial

Reiko Tanihiro [1,*], Masahiro Yuki [1], Katsuhisa Sakano [1], Masaki Sasai [1], Daisuke Sawada [1], Shukuko Ebihara [2] and Tatsuhiko Hirota [1]

1 Core Technology Laboratories, Asahi Quality and Innovations, Ltd., Moriya 302-0106, Japan

2 Chiyoda Paramedical Care Clinic, Tokyo 101-0047, Japan

* Correspondence: reiko.tanihiro@asahi-qi.co.jp; Tel.: +81-297-46-9347

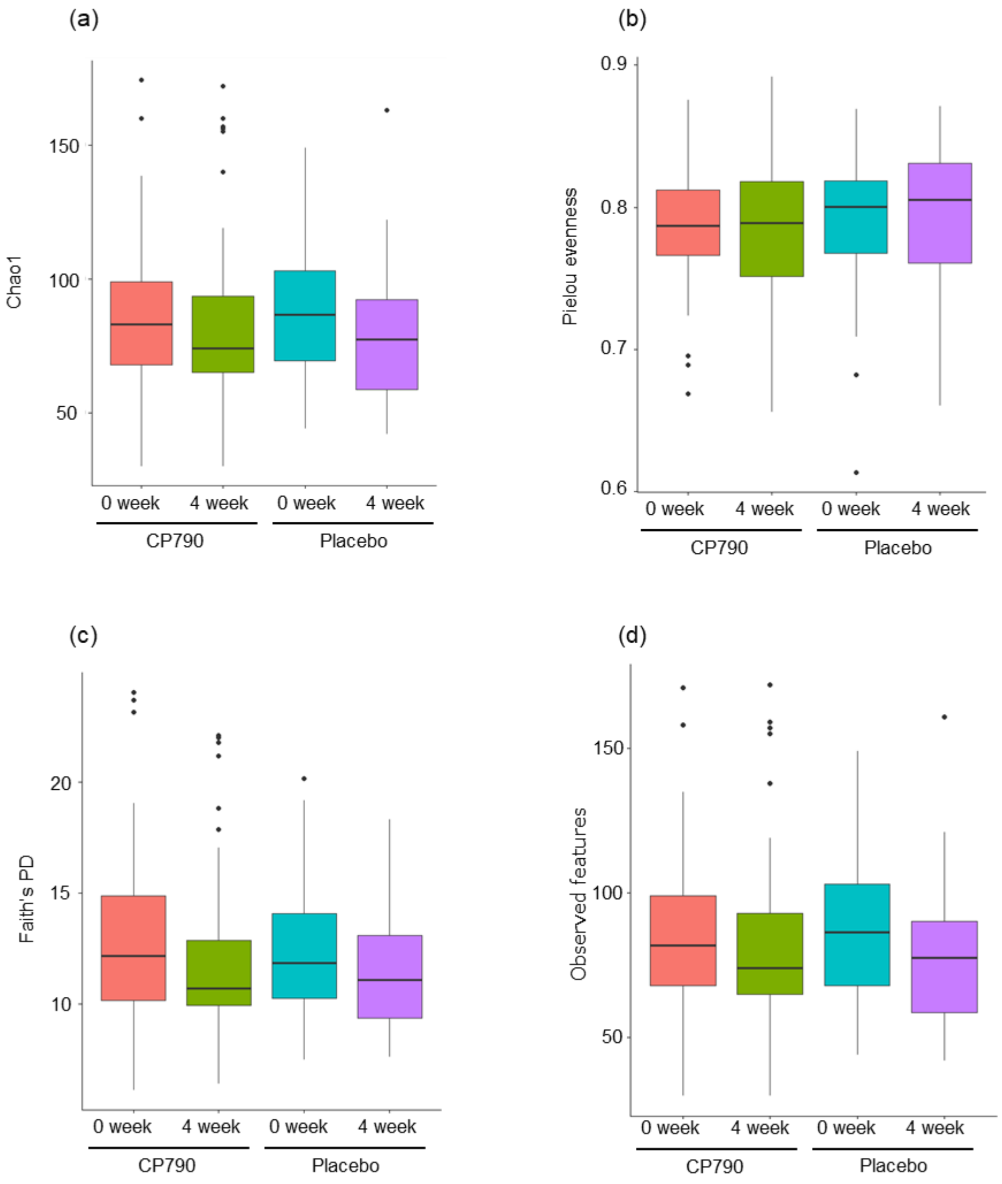

Figure S1. Alpha diversity metrics: (a) Chao1, (b) Pielou's Evenness, (c) Faith's PD, and (d) Observed features.

Table S1. Defecation habits.

Items	Baseline				Post-intervention			
	CP790 (n = 57)		Placebo (n = 60)		CP790 (n = 57)		Placebo (n = 60)	
	Mean	SE[1]	Mean	SE[1]	Mean	SE[1]	Mean	SE[1]
Defecation frequency (times/week)	4.35	0.13	4.36	0.15	5.39	0.23	5.37	0.21
Bristol Scale Scores	3.48	0.09	3.49	0.09	3.86	0.07 *	3.64	0.08
Straining during defecation	2.11	0.08	1.90	0.10	1.82	0.08	1.79	0.09
Feeling of incomplete evacuation	2.06	0.08	1.84	0.10	1.79	0.08	1.70	0.09

[1] SE: standard error. Differences between groups were compared using the Student's t-test (* $p < 0.05$).

Table S2. PAC-SYM scores

Items	Baseline				Post-intervention			
	CP790 (n = 57)		Placebo (n = 60)		CP790 (n = 57)		Placebo (n = 60)	
	Mean	SE[1]	Mean	SE[1]	Mean	SE[1]	Mean	SE[1]
Abdominal symptoms	0.30	0.05	0.30	0.05	0.16	0.05	0.24	0.05
Rectal symptoms	0.16	0.05	0.23	0.06	0.06	0.02	0.08	0.03
Stool symptoms	0.77	0.10	0.58	0.09	0.48	0.08	0.39	0.07
Total PAC-SYM	0.46	0.06	0.40	0.06	0.27	0.05	0.26	0.05

[1] SE: standard error. Differences between groups were compared using the Mann–Whitney U-test.

Table S3. Spearman's correlation coefficients.

	Defecation frequency	BSS	Straining	Incomplete evacuation	*Desulfobacterota*	*Actinobacteriota*	*Firmicutes*	*Bacteroidota*	*Proteobacteria*
Defecation frequency		0.174	−0.213 *	−0.100	−0.058	−0.022	0.009	0.028	−0.054
BSS			−0.681 *	−0.557 *	−0.188 *	−0.113	0.020	−0.054	0.085
Straining				0.653 *	−0.187 *	0.031	−0.089	0.114	−0.038
Incomplete evacuation					0.225 *	0.124	−0.078	0.096	−0.066
Desulfobacterota						0.209 *	−0.113	0.065	0.096
Actinobacteriota							0.264 *	−0.331 *	0.078
Firmicutes								−0.723 *	−0.197 *
Bacteroidota									−0.252 *
Proteobacteria									

Significant difference at * $p < 0.05$.
